# Geographic Variation of Overweight and Obesity among Women in Nigeria: A Case for Nutritional Transition in Sub-Saharan Africa

**DOI:** 10.1371/journal.pone.0101103

**Published:** 2014-06-30

**Authors:** Ngianga-Bakwin Kandala, Saverio Stranges

**Affiliations:** 1 Division of Health Sciences, University of Warwick Medical School, Coventry, United Kingdom; 2 Epidemiology and Biostatistics Division, School of Public Health, University of the Witwatersrand, Johannesburg, South Africa; 3 University of Oxford, KEMRI-University of Oxford-Wellcome Trust Collaborative Programme, Malaria Public Health and Epidemiology Group, Centre for Geographic Medicine, Nairobi, Kenya; Chancellor College, University of Malawi, Malawi

## Abstract

**Background:**

Nutritional research in sub-Saharan Africa has primarily focused on under-nutrition. However, there is evidence of an ongoing nutritional transition in these settings. This study aimed to examine the geographic variation of overweight and obesity prevalence at the state-level among women in Nigeria, while accounting for individual-level risk factors.

**Methods:**

The analysis was based on the 2008 Nigerian Demographic and Health Survey (NDHS), including 27,967 women aged 15–49 years. Individual data were collected on socio-demographics, but were aggregated to the country's states. We used a Bayesian geo-additive mixed model to map the geographic distribution of overweight and obesity at the state-level, accounting for individual-level risk factors.

**Results:**

The overall prevalence of combined overweight and obesity (body mass index ≥25) was 20.9%. In multivariate Bayesian geo-additive models, higher education [odds ratio (OR) & 95% Credible Region (CR): 1.68 (1.38, 2.00)], higher wealth index [3.45 (2.98, 4.05)], living in urban settings [1.24 (1.14, 1.36)] and increasing age were all significantly associated with a higher prevalence of overweight/obesity. There was also a striking variation in overweight/obesity prevalence across ethnic groups and state of residence, the highest being in Cross River State, in south-eastern Nigeria [2.32 (1.62, 3.40)], the lowest in Osun State in south-western Nigeria [0.48 (0.36, 0.61)].

**Conclusions:**

This study suggests distinct geographic patterns in the combined prevalence of overweight and obesity among Nigerian women, as well as the role of demographic, socio-economic and environmental factors in the ongoing nutritional transition in these settings.

## Introduction

Nutritional research in sub-Saharan Africa has primarily focused on under-nutrition, particularly among vulnerable population subgroups such as women and children. However, while sub-Saharan Africa is still one of the poorest regions in the world with extremely high rates of malnutrition, there is suggestive evidence of an ongoing nutritional transition [1]–[3]. Data from several developing countries suggest that rising urbanisation and improvements in developmental indicators lead to concurrent under- and over-nutrition in the population [4]–[7].

Furthermore, in these settings the nutritional transition often takes place in an environment which has traditionally favoured ‘plumpness’ as a symbol of sexual beauty, socioeconomic status and social standing [8]–[9]. When preference for large body shapes exists, a direct relationship between socioeconomic status and obesity is likely to be observed at the beginning of the transition, especially in women, because higher socioeconomic groups have the financial capacity to buy extra food and achieve their desire to look healthy and strong [9].

Although the growing number of obese individuals has received attention in many developing countries, sub-Saharan Africa is still lacking research into this subject partially due to the persisting high proportion of the population classified as underweight. Nonetheless, the ongoing nutritional transition in these settings has been mostly linked to the rapid process of urbanisation and westernisation [1]–[7]. In 1995, 35% of the African population lived in urban areas, whereas 54% of the population is projected to be urban by 2030, with estimates ranging from 43% in East Africa to 67% in southern Africa [10].

Furthermore, in the last decades the number of deaths from non-communicable diseases in developing countries has already exceeded those observed in developed countries [11]–[12]. Moreover, obesity is associated with an increased risk for many of these chronic conditions including heart disease, hypertension, arthritis and diabetes mellitus [13]–[15].

The ongoing nutritional transition in Africa is likely to pose a major public health challenge with a significant proportion of adults becoming overweight, whilst large segments of the population still face risk of morbidity and mortality related to under-nutrition [16].

Understanding the underlying ecological and socioeconomic roots of both extremes of the nutritional status is vital to designing successful interventions and targeting limited resources to the dual problem of under- and overweight in African settings.

The aim of this study was to examine the geographic variation of overweight/obesity at the state-level among women in Nigeria, using the 2008 Nigerian Demographic and Health Survey (NDHS) data [17]. We also examined a wide range of socioeconomic, individual and household factors which are likely to affect the ongoing nutritional transition in these settings.

## Methods

### Study Population

The Demographic and Health Survey (DHS), funded by the United States Agency for International Development (USAID), is a well-established source of reliable population level data with a substantial focus on health. The objectives, organisation, sample design and questionnaires used in the DHS surveys are described elsewhere [17]. This study was approved by the relevant institutional review board for DHS surveys. The authors thank Macro international for providing free access to the 2008 DHS datasets for Nigeria. Data were handled in an anonymized fashion.

A random sample of 34,596 women were identified to be eligible for the individual interview, anthropometric measurements of height and weight were collected for women aged 15–49 years, and risk factors for obesity were recorded, as were socio-demographic data and the state of residence. The body mass index (BMI), or the Quetelet index, was used to measure thinness and obesity. There were few participants with missing data for BMI, and other covariates; thus, data analysis on BMI was based on 27,967 women with a complete set of data [17].

### Outcome Measurement

Anthropometric measures were taken, including height, weight, and waist circumference; body mass index (BMI) was calculated as weight in kilograms divided by height in meters squared and categorized as follows: underweight, BMI <18.5 kg/m^2^; normal weight, BMI 18.5–24.9 kg/m^2^ (reference category); overweight, BMI 25–29.9 kg/m^2^; and obese, BMI ≥30 kg/m^2^, according to WHO guidelines [18]. For the present analyses, the outcome or dependent variable was combined prevalence of overweight and obesity, defined as BMI ≥25 kg/m^2^, as compared to normal weight, defined as BMI 18.5–24.9 kg/m^2^ (reference category). We choose the binary outcome instead of the continuous BMI values, due to interpretability reasons since with the binary outcome one can estimate the likelihood of overweight/obesity in a given state, while accounting for a number of potential covariates.

### Covariates

**Figure 1:**
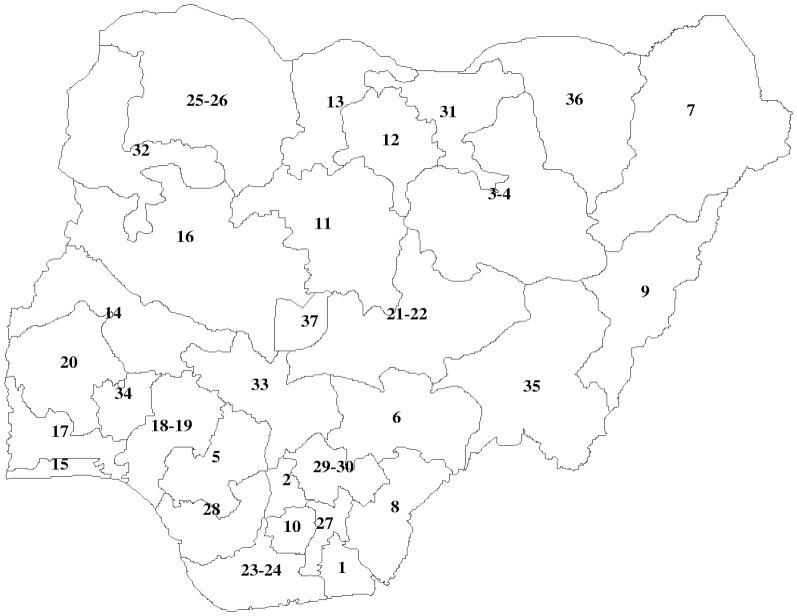
Map of Nigeria by states. 1, Akwa Ibom. 2, Anambra. 3, Bauchi. 4, Gombe. 5, Edo. 6, Benue. 7, Borno.-River. 9, Adamawa. 10, Imo. 11, Kaduna. 12, Kano. 13, Katsina. 14, Kwara. 15, Lagos. 16, Niger. 17, Ogun. 18, Ondo. 19, Ekiti. 20, Oyo. 21, Nassarawa. 22, Plateau. 23, Bayelsa. 24, Rivers. 25, Sokoto. 26, Zamfara. 27, Abia. 28, Delta. 29, Ebonyi. 30, Enugu. 31, Jigawa. 32, Kebbi. 33, Kogi. 34, Osun. 35, Taraba. 36, Yobe. 37, Abuja.

The main exposure variable investigated was the respondent's geographic location, i.e. the state of residence at the time of the survey (**figure 1**), in addition to various individual-level control variables such as socio-demographics known to be associated with nutritional status. The respondent and her partner's age at the time of survey were also included as an indicator of the birth cohort of the women. Other socio-demographic covariates were religion (catholic versus other Christian, Islam, traditionalist and other), wealth index (poorest versus poorer, middle, richer and richest), and education of the respondent and partner (no education vs. primary, secondary and higher education). Finally, environmental factors included place (locality) of residence (rural vs. urban) and state of residence of the women including her ethnicity (Ekoi versus Fulani, Hausa, Ibibio, Igala, Igbo, Ijaw/Izon, Kanuri/Beriberi, Tiv, Yoruba and others). It should be noted that Nigeria is currently divided into 36 states and Abuja, the federal capital territory, while it was previously divided into 30 states and Abuja (1991–1996), with the further addition of six states in 1996.

### Statistical Analysis

To account for spatial effects in the prevalence of combined overweight/obesity at the state level, in Nigeria, we applied a unified approach to account for possible nonlinear effects of continuous risk factors. This was achieved using a geo-additive semi-parametric mixed model. The model employed a fully Bayesian approach using Markov Chain Monte Carlo (MCMC) techniques for inference and model checking [19]–[21]. The response variable was defined as *y_i_ = 1 if overweight/obese and y_i_ = 0 normal weight*. The standard measure of effect was the posterior odds ratio (POR) and 95% credible region (CR).

The analysis was carried out using version 2.0.1 of the BayesX software package, which permits Bayesian inference based on Markov chain Monte Carlo (MCMC) simulation techniques [22]. The statistical significance of apparent associations between potential risk factors and the prevalence of overweight/obesity was explored with chi-square (χ^2^) and Mann–Whitney *U*-tests, as appropriate. Adjusted marginal ORs of overweight/obesity risk across states were obtained from standard logistic regression models, with Yobe used as the reference category because of the lowest crude overweight/obesity prevalence. Multivariate Bayesian geo-additive regression models were used to evaluate the significance of the posterior OR determined for the fixed, non-linear effects and spatial effects.

## Results

Baseline characteristics of the study population are displayed in **Table 1** for the overall sample (N = 27,967), and in **Table 2** with participants split within the two categories of overweight/obese vs. normal-weight status (N = 24,614), after excluding underweight subjects (N = 3,353). Overall, mean age of participants was 29.3 (±7.1) and for their partners 40.9 (±12.8). The percentage of participants with no education was high (50.3%) and 41.4% among their partners. Mean BMI was 22.0 (±4.3); 26.6% of participants were living in an urban area, 56.7% were Muslim and 26.5% were in the poorest quintile of the wealth index. Prevalence values of underweight, normal-weight, overweight and obese were: 12.0%, 67.1%, 15.7% and 5.2%, respectively. The combined overweight/obesity prevalence was 20.9%, with variation across states ranging from 10.5% in Yobe to 50.2% in Lagos.

**Table 1:** Baseline characteristics of the study population (NDHS, 2008)*.

Variable	N = 27,967
Mean age† (SD) for respondent	29.3(7.1)
Mean age‡ (SD) for partner	40.9(12.8)
Education respondent (%)	
No education	50.3
Primary education	22.9
Secondary education	22.1
Higher education	4.7
Education partner (%)	
No education	41.4
Primary education	21.4
Secondary education	26.4
Higher education	10.8
Urban population (%)	26.6
Mean BMI, kg/m^2^ (SD)	22.0(4.3)
Religion (%)	
Catholic	8.6
Other Christian	32.6
Islam	56.7
Traditionalist	1.9
Other	0.1
Wealth Index (%)	
Poorest	26.5
Poorer	24.0
Middle	19.6
Richer	16.6
Richest	13.3
BMI categories (%)	
Underweight (BMI<18.5)	12.0
Normal weight (BMI 18.5–24.9)	67.1
Overweight (BMI 25–29.9)	15.7
Obese (BMI ≥30)	5.2
Ethnicity (%)	
Ekoi	1.5
Fulani	9.6
Hausa	28.2
Ibibio	1.6
Igala	1.2
Igbo	10.5
Ijaw/Izon	3.1
Kanuri/Beriberi	3.3
Tiv	2.8
Yoruba	10.8
Others	27.5
State of residence (%)	
Akwa Ibom	1.8
Anambra	1.7
Bauchi/Gombe	8.1
Edo	2.2
Benue	3.0
Borno	4.0
Crossriver	2.0
Adamawa	3.4
Imo	1.4
Kaduna	3.5
Kano	5.1
Katsina	4.7
Kwara	1.9
Lagos	2.6
Niger	3.7
Ogun	1.8
Ondo/Ekiti	3.6
Oye	1.9
Nassarawa/Plateau	5.1
Rivers/Bayelsa	3.8
Sokoto/Zamfara	7.3
Abia	1.6
Delta	1.8
Enungu/Ebonyi	3.9
Jigawa	3.9
Kebbi	3.3
Kogi	1.9
Osun	1.7
Taraba	3.5
Yobe	3.9
Abuja	2.1

* Data are expressed as mean (standard deviation) or as percentages.

† Age ranges from 15 to 49 years of age.

‡ Age ranges from 15 to 95 years of age.

**Table 2:** Baseline characteristics of the study population by overweight/obesity status (NDHS 2008)*.

Variable	Overweight/Obese (N = 5,836)	Normal-weight (N = 18,778)	P-value†
Mean age (SD) for respondent	31.3(6.6)	28.9(7.1)	P<0.001
Mean age (SD) for partner	42.2(11.9)	40.5(12.9)	P<0.001
Education respondent			P<0.001
No education	1808(15.7)	9730(84.3)	
Primary education	1369(23.2)	4543(76.8)	
Secondary education	2009(34.1)	3881(65.9)	
Higher education	650(51.0)	624(49.0)	
Education partner			P<0.001
No education	1472(16.2)	7624(83.8)	
Primary education	1210(23.0)	4048(77.0)	
Secondary education	1874(28.0)	4813(72.0)	
Higher education	1124(40.7)	1640(59.3)	
Place of residence			P<0.001
Urban	2500(36.4)	4365(63.6)	
Rural	3336(18.8)	14413(81.2)	
Religion			P<0.001
Catholic	724(31.5)	1577(68.5)	
Other Christian	2604(30.1)	6045(69.9)	
Islam	2372(18.2)	10662(81.8)	
Traditionalist	88(19.6)	360(80.4)	
Other	14(38.9)	22(61.1)	
Wealth Index			P<0.001
Poorest	718(12.0)	5279(88.0)	
Poorer	828(14.4)	4920(85.6)	
Middle	1195(24.2)	3745(75.8)	
Richer	1386(32.0)	2943(68.0)	
Richest	1709(47.5)	1891(52.5)	
Ethnicity			P<0.001
Ekoi	67(16.7)	334(83.3)	
Fulani	202(10.2)	1787(89.8)	
Hausa	1120(17.5)	5275(82.5)	
Ibibio	153(36.8)	263(63.2)	
Igala	99(31.5)	215(68.5)	
Igbo	1091(39.0)	1708(61.0)	
Ijaw/Izon	297(35.3)	544(64.7)	
Kanuri/Beriberi	115(16.5)	584(83.5)	
Tiv	102(14.0)	626(86.0)	
Yoruba	879(31.3)	1934(68.7)	
Others	1684(23.8)	5400(76.2)	
State of residence			P<0.001
Akwa Ibom	169(34.9)	316(65.2)	
Anambra	222(47.2)	248(52.8)	
Bauchi/Gombe	213(12.1)	1544(87.9)	
Edo	230 (39.2)	357 (60.8)	
Benue	92(11.9)	683(88.1)	
Borno	193(21.8)	693(78.2)	
Cross River	133(24.2)	417(75.8)	
Adamawa	136(16.1)	709(83.9)	
Imo	183(46.8)	208(53.2)	
Kaduna	196(22.2)	688(77.8)	
Kano	227(19.1)	963(80.9)	
Katsina	113(10.8)	929(89.2)	
Kwara	118(24.1)	372(75.9)	
Lagos	357(50.2)	354(49.8)	
Niger	199(20.7)	762(79.3)	
Ogun	98(21.6)	355(78.4)	
Ondo/Ekiti	254(26.9)	690(73.1)	
Oye	135(26.7)	370(73.3)	
Nassarawa/Plateau	276(20.3)	1083(79.7)	
Rivers/Bayelsa	410(39.4)	631(60.6)	
Sokoto/Zamfara	230(14.8)	1323(85.2)	
Abia	158(38.6)	251(61.4)	
Delta	123(26.9)	335(73.1)	
Enungu/Ebonyi	253(25.5)	741(74.5)	
Jigawa	123(13.9)	760(86.1)	
Kebbi	207(26.9)	563(73.1)	
Kogi	141(28.6)	352(71.4)	
Osun	82(19.3)	343(80.7)	
Taraba	238(26.1)	674(73.9)	
Yobe	87(10.5)	741(89.5)	
Abuja	240(42.6)	323(57.4)	

*Data are expressed as mean (standard deviation) or as percentages.

† *P*-values for comparison between overweight/obese and normal weight subjects.

On average, overweight/obese respondents were older than their normal-weight counterparts, were more likely to be highly educated and have highly educated partners, more likely to be living in urban areas and more likely to be from other religions. In addition, overweight/obese women were more likely to be in the richest quintile of the wealth index, more likely to be from the Igbo ethnic group and living in Lagos State.

Associations were in the opposite directions when comparing underweight women with normal-weight subjects (**Table S1**). In fact, underweight respondents were younger than their normal-weight counterparts, were more likely to have no education and have uneducated partners, more likely to be living in rural settings and more likely to be either Muslim or traditionalist. In addition, underweight women were more likely to be in the poorest quintile of the wealth index, more likely to be from the Kanuri/Beriberi ethnic group and living in Yobe State, an agricultural state and one of the poorest regions in Nigeria.

**Figure 2:**
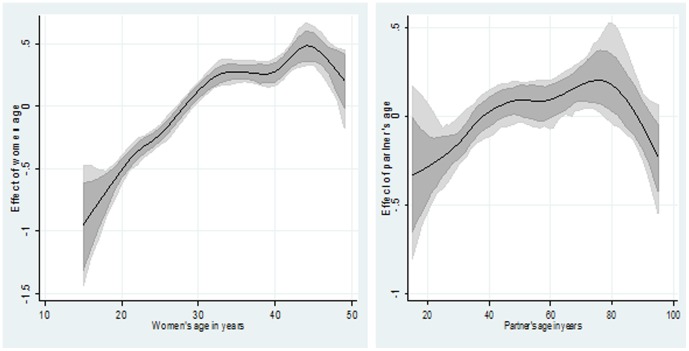
Estimated nonparametric effect of respondent (left) and partner's (right) age on obesity/overweight. Shown is the posterior mean within 80% credible regions.

**Table 3** displays both marginal and posterior odds ratios of overweight/obese across the selected study characteristics. Results from both standard logistic regression and multivariate Bayesian geo-additive analyses (right-hand column) support the role of women's wealth, higher education and living in an urban setting as risk factors. Specifically, women with higher education [posterior odds ratio (POR) & 95% credible region (CR): 1.68 (1.38, 2.00)] from the richest wealth index [3.45 (2.98, 4.05)] were consistently associated with higher odds of being overweight/obese. Also women from the Igala ethnic group [7.47 (3.98, 12.3)] were consistently associated with higher odds of being overweight/obese. The associations of partner education and religion with overweight/obesity risk were not statistically significant. Moreover, there were nonlinear associations between women and partner's age and overweight/obesity status: the risk of overweight/obesity increased with age with a peak at around 45 years of age for women and 70 years of age for their partners and then a decrease thereafter (**Figure 2** **)** using a flexible nonlinear curve.

**Table 3:** Marginal and posterior odds ratios of overweight/obesity across selected covariates (NDHS, 2008).

Variable	Marginal OR & 95%CI‡	Posterior OR & 95% CI†
Age groups of respondent		
< = 25	1.00	See Figure 2 left
26–35	1.55(1.41,1.70)	
36–49	1.85(1.65,2.08)	
Age groups of partner		
< = 30	1.00	See Figure 2 right
31–40	1.29(1.15,1.43)	
41+	1.45(1.29,1.64)	
Education respondent		
No education	1.00	1.00
Primary education	1.15(1.03, 1.28)	1.17(1.04, 1.32)
Secondary education	1.43(1.27, 1.62)	1.49(1.32, 1.73)
Higher education	1.64(1.37, 1.96)	1.68(1.38, 2.00)
Education partner		
No education	1.00	1.00
Primary education	0.84(0.75, 0.93)	0.83(0.76, 0.93)
Secondary education	0.80(0.71, 0.89)	0.81(0.72, 0.91)
Higher education	0.96(0.84, 1.10)	0.96(0.85, 1.13)
Place of residence		
Urban	1.24(1.14,1.35)	1.24(1.14, 1.36)
Rural	1.00	1.00
Religion		
Catholic	1.00	1.00
Other Christian	0.77(0.68, 0.88)	0.77(0.68, 0.89)
Islam	0.73(0.62, 0.86)	0.74(0.63, 0.91)
Traditionalist	0.73(0.55, 0.97)	0.75(0.58, 0.97)
Other	1.39(0.68, 2.83)	1.49(0.68, 2.79)
Wealth Index		
Poorest	1.00	1.00
Poorer	1.19(1.06, 1.34)	1.19(1.06, 1.31)
Middle	1.91(1.70, 2.15)	1.89(1.67, 2.13)
Richer	2.48(2.17, 2.83)	2.44(2.08, 2.82)
Richest	3.55(3.03, 4.15)	3.45(2.98, 4.05)
Ethnicity		
Ekoi	1.00	1.00
Fulani	3.28(1.93, 5.57)	3.10(1.81, 5.54)
Hausa	4.54(2.72, 7.59)	4.29(2.69, 7.12)
Ibibio	4.65(2.81, 7.71)	4.79(2.77, 8.47)
Igala	8.15(4.52, 14.7)	7.47(3.98, 12.3)
Igbo	7.03(4.22, 11.7)	6.41(4.19, 10.4)
Ijaw/Izon	7.11(4.08, 12.4)	6.22(3.97, 10.4)
Kanuri/Beriberi	3.73(2.13, 6.52)	3.47(1.92, 6.22)
Tiv	5.62(3.14, 10.1)	4.53(2.43, 8.45)
Yoruba	6.28(3.74, 10.6)	5.44(3.27, 9.18)
Others	5.12(3.13, 8.40)	4.78(2.99, 7.94)
State of residence		
Akwa Ibom	2.41(1.66, 3.50)	1.50(1.16, 1.89)
Anambra	1.53(1.07, 2.19)	1.02(0.78, 1.33)
Bauchi/Gombe	1.29(0.98, 1.70)	0.82(0.68, 0.97)
Edo	1.89(1.39, 2.58)	1.20(0.97, 1.48)
Benue	0.76(0.51, 1.14)	0.53(0.38, 0.68)
Borno	2.25(1.68, 3.03)	1.43(1.13, 1.75)
Cross River	4.49(2.80, 7.19)	2.32(1.62, 3.40)
Adamawa	1.43(1.05, 1.94)	0.98(0.78, 1.17)
Imo	2.05(1.41, 2.98)	1.37(1.03, 2.00)
Kaduna	1.68(1.25, 2.25)	1.09(0.91, 1.32)
Kano	1.67(1.25, 2.23)	1.06(0.88, 1.30)
Katsina	1.07(0.78, 1.48)	0.71(0.55, 0.87)
Kwara	1.26(0.89, 1.78)	0.82(0.60, 1.04)
Lagos	1.68(1.23, 2.30)	1.14(0.94, 1.37)
Niger	1.73(1.30, 2.31)	1.11(0.93, 1.32)
Ogun	0.89(0.62, 1.28)	0.64(0.49, 0.84)
Ondo/Ekiti	1.16(0.84, 1.60)	0.76(0.61, 0.90)
Oye	1.08(0.76, 1.54)	0.74(0.59, 0.97)
Nassarawa/Plateau	1.51(1.14, 1.99)	0.95(0.78, 1.11)
Rivers/Bayelsa	2.18(1.54, 3.09)	1.43(1.15, 1.87)
Sokoto/Zamfara	1.52(1.14, 2.03)	0.98(0.81 1.18)
Abia	1.34(0.93, 1.94)	0.96(0.72, 1.25)
Delta	1.26(0.90, 1.78)	0.86(0.67, 1.07)
Enungu/Ebonyi	1.07(0.76, 1.50)	0.72(0.57, 0.95)
Jigawa	1.50(1.09, 2.06)	0.92(0.73, 1.12)
Kebbi	3.32(2.48, 4.44)	2.05(1.65, 2.60)
Kogi	1.29(0.90, 1.84)	0.81(0.63, 1.03)
Osun	0.64(0.44, 0.95)	0.48(0.36, 0.61)
Taraba	2.74(2.06, 3.64)	1.72(1.42, 2.06)
Yobe	1.00	1.00
Abuja	1.90(1.40, 2.58)	1.24(0.99, 1.47)

‡ Adjusted marginal odds ratio (OR) from standard logistic regression models.

† Spatially adjusted posterior odds ratio (OR) from Bayesian geo-additive regression models after controlling for nonlinear effect of age, categorical variables and the province of residence (spatial effects).

With regard to overweight/obesity status, in the marginal regression analyses there was a striking variation in overweight/obesity risk across states, the highest being in Cross River State [4.49 (2.80, 7.19)], Kebbi [3.32 (2.48, 4.44)] and Taraba [2.74 (2.06, 3.64)], the lowest in Osun [0.64 (0.44, 0.95)] and Benue [0.76 (0.51, 1.14)].

**Figure 3:**
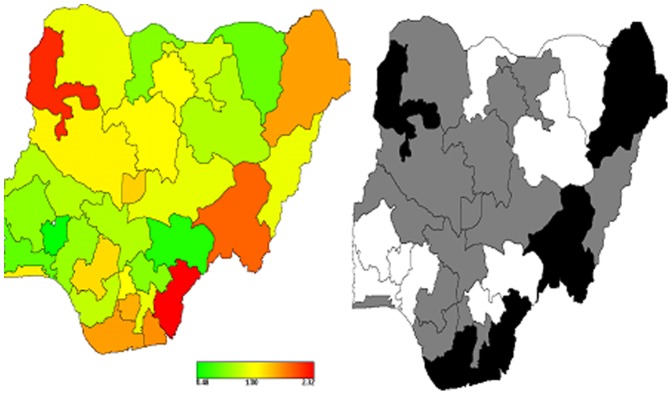
Total residual spatial effects, at state level in Nigeria, of obesity/overweight. Shown are the posterior odds ratios (left) and corresponding posterior probabilities at 80% nominal level (right).

**Figure 3** shows results for covariate-adjusted state overweight/obesity status spatial variation captured by the global total state effects. There was a clear pattern of states with higher risk of overweight/obesity, mostly the south-eastern states of Cross River, Akwa Ibom, Rivers and Bayelsa, including the eastern state of Taraba and the northern state of Kebbi, which were associated with higher prevalence of overweight/obesity, while states in the west and north were associated with a lower overweight/obesity prevalence. These spatial patterns confirm the observed marginal model findings shown in Table 3.

Specifically, the left-hand map shows estimated posterior total residual state odds of overweight/obesity for each state, ranging from a lower POR of 0.48 (0.36, 0.61) in Osun State to a higher POR of 2.32 (1.62, 3.40) in Cross River State, with red colour indicating the higher risk recorded and green colour denoting lower odds. The right-hand map shows the 95% posterior probability map of overweight/obesity, which indicates the statistical significance associated with the total excess risk. White indicates a negative spatial effect (associated with reduced risk of overweight/obesity), black a positive effect (an increased risk) and grey a non-significant effect. However, the total spatial residuals in Figure 3 show that much of the variation in overweight/obesity remains to be explained.

The spatial effects of the populous Lagos State was greatly attenuated after multiple adjustments of the urban environment and other risk factors indicating that perhaps the higher number of obese/overweight women living in Lagos was inflated by the large number of women living in Lagos and the younger age structure of urban areas.

## Discussion

To our knowledge, this is the first and largest epidemiological study which examined the geographic variation, at the state-level, of combined prevalence of overweight and obesity in an adult population from sub-Saharan Africa. We used data from the 2008 Nigerian DHS on a large, nationwide sample of women across the country's states. Overall, the combined prevalence of overweight and obesity was 20.9%, with a striking variation across ethnic groups and states of residence, ranging from 10.5% in Yobe to 50.2% in Lagos. Findings also point to the crucial role of demographic, socio-economic and environmental factors driving the ongoing nutritional transition in these settings. In fact, we found that women in older age groups, with higher education and wealth index, and living in urban settings were all significantly associated with a higher prevalence of combined overweight and obesity.

These findings provide novel evidence to support the notion of a rapidly changing scenario in sub-Saharan Africa, in terms of nutrition and dietary patterns, with co-existence of a dual burden of under- and overnutrition, which is already posing a major public health challenge in these low-resource settings [14]–[16]. Several factors are likely to contribute to the ongoing nutritional transition in these countries, primarily the rapid process of urbanisation and westernisation, with over-reliance on energy-dense processed foods, decreased intakes of staple and low-calorie foods, physical inactivity and sedentary lifestyles [23]–[24]. The rapidly changing role of women in these societies, with their increasing involvement in the labour force especially in urban areas, might also contribute to the dramatic changes in dietary patterns and food supply occurring in these settings [25]. However, we cannot disregard the role of cultural factors in this transition, because in developing societies ‘fatness’ has been often considered as a symbol of sexual beauty and social standing, particularly among women. In addition, in these settings a direct relationship between socioeconomic status and obesity has been observed, since higher socioeconomic groups are more likely to buy extra food and achieve their desire to look healthy and strong [8]–[9].

Our estimates of overweight and obesity prevalence are generally in keeping with previous studies from Nigeria [26]–[33], as well as with WHO reports on Sub-Saharan African countries [34]. Specifically, in the latest published systematic review, which included only four good-quality communitywide studies in Nigeria, Chukwuonye *et al* found that the prevalence of overweight ranged from 20.3% to 35.1%, while the prevalence of obesity ranged from 8.1% to 22.2% [32]. The four included studies comprised participants from both sexes and showed a consistently higher prevalence of obesity among women, which is in line with the notion that the ongoing nutritional transition in sub-Saharan Africa, and possibly in other developing countries, may affect primarily this population subgroup due to a range of demographic, cultural, socio-economic and environmental factors [14]–[16].

These observations have been further corroborated by a recent cross-sectional study on the prevalence of abdominal obesity in Abia State, south-eastern Nigeria, among 2,807 individuals of both sexes [33]. In this study, abdominal obesity was defined as a waist circumference of 102 cm or more in men and 88 cm or more in women. While the prevalence of general obesity (BMI≥30) was twice as high in women than men (14.37% vs. 7.73%, respectively), there was a much wider gap between the two sexes in the prevalence of abdominal obesity, being 39.2% among women and only 3.2% in men. Again, biological and experimental evidence on potential mechanisms that may explain this remarkable sex difference in body fat distribution in the Sub-Saharan African context is extremely limited; therefore any such discussion is highly speculative. However, it is believed that, in these societies, men still engage in more strenuous physical activities than women [33]. An additional plausible explanation may reside in the fact that women in developing countries commonly undergo multiple pregnancies, which is a potential risk factor for female obesity and central adiposity [35].

It should be noted that in our study the prevalence estimates of both overweight (15.7%) and obesity (5.2) were somewhat lower than those reported in some previous Nigerian surveys [26]–[33]. However, the mean age of participants in our sample was 29.3 (age range: 15–49), whereas previous studies were generally based on samples of middle-aged individuals, among whom the prevalence of both overweight and obesity is expected to be higher. In fact, in our sample of women the risk of overweight/obesity increased with age with a peak at around 45 years of age.

Unlike previous surveys from Nigeria [26]–[33], this is the first study that attempted to estimate the geographic variation, at the state-level, of combined prevalence of overweight and obesity in an adult population from sub-Saharan Africa, independent of individual-level risk factors. Our findings point to a striking variation in overweight/obesity prevalence across states of residence. Specifically, in *unadjusted analyses* there was a clear pattern of higher prevalence of overweight/obesity in those states with higher degrees of urbanization, namely Lagos State (50.2%), which covers the most populous area in Nigeria and one of the fastest growing cities in the world [36]. On the other hand, the prevalence of overweight/obesity was the lowest in Yobe State (10.5%), in Northern Nigeria, which mainly covers an agricultural area. However, in multivariate Bayesian geo-additive regression models, there was evidence of a slightly different scenario. Specifically, there was a clear pattern of states with higher risk of overweight/obesity in the south-eastern states of Cross River, Akwa Ibom, Rivers, Bayelsa, and Taraba, whereas states in the west and north were generally associated with lower overweight/obesity prevalence (except for the northern state of Kebbi). Importantly, the elevated unadjusted prevalence of overweight/obesity of the populous Lagos State was greatly attenuated after multiple adjustments for the urban environment and other risk factors, suggesting that the unadjusted estimate was possibly inflated by the large number of women living in Lagos and the younger age structure of population in urban settings.

Potential explanations for the observed geographic variation in the prevalence of overweight/obesity across the country's states are highly speculative at the present time, given the lack of published literature or policy reports in this specific area of research. This geographic variation could well be due to differences in lifestyles, level of urbanisation and westernisation, wealth distribution, access to food supply and increasing presence of fast food chains and outlets in these states [37]–[38]. For example, Cross River State and its capital Calabar are now among the leading tourism sites of Nigeria, where the large presence of international visitors is likely to influence local lifestyles and dietary patterns, with a gradual process of westernisation, penetration of the fast food sector, increasing intake of energy-dense processed foods, and related health consequences, such as obesity [39].

There are some limitations in the present study that deserve discussion. First, the cross-sectional nature of the present study does not allow for establishing temporality and causality of the observed associations. However, our exposure variables were primarily demographic and socioeconomic factors, whereby the potential of reverse causation should be minimized. Second, the analysis was based on a nationally representative sample of Nigerian women; therefore, the generalizability and applicability of these findings to male populations or other Sub-Saharan African countries warrants further investigations. Finally, there was limited or lack of information for variables such as dietary habits, physical activity, and biomarker data, which are relevant to metabolism and overweight/obesity aetiology. Therefore, we cannot rule out the possibility that our findings might have been somewhat biased by the lack of these important confounders.

In conclusion, this is the first and largest study that examined the geographic variation of combined prevalence of overweight and obesity in an adult population from sub-Saharan Africa. We found several consistent associations between socio-demographic variables and prevalence of overweight/obesity in a nationwide sample of Nigerian women from the 2008 DHS. The geographic analysis showed distinct patterns in the prevalence of overweight/obesity across the country's states, pointing to the potential influence of demographic, cultural, socio-economic and environmental factors, as well as to an increasing level of urbanisation and westernisation, which are all driving the ongoing nutritional transition in these settings. Importantly, policy makers and public health practitioners can use this geographic information on overweight/obesity mapping for planning purposes, educational and nutritional programs on lifestyles and dietary patterns, but also in the decision making process for the allocation of public resources to the most affected areas of the population, especially in these low-income settings [40].

## Supporting Information

Table S1 **Baseline characteristics of the study population by underweight status (NDHS 2008).**
(DOC)
