# Supplementary material for: Geographic Variation of Overweight and Obesity among Women in Nigeria: A Case for Nutritional Transition in Sub-Saharan Africa
Source: PLoS One. 2014 Jun 30;9(6):e101103. doi: 10.1371/journal.pone.0101103 (PMC4076212; doi:10.1371/journal.pone.0101103)
Supplement: Table S1 — Baseline characteristics of the study population by underweight status (NDHS 2008). (DOC) [file pone.0101103.s001.doc]

**Supplementary Table 1: Baseline characteristics of the study population by underweight status** **(NDHS 2008)***

| **Variable** | Underweight  (N=3,353) | Normal weight (N=18,778) | P-value^†^ |
| --- | --- | --- | --- |
| Mean age (SD) for respondent | 28.1(7.0) | 28.9(7.1) | P<0.001 |
| Mean age (SD) for partner | 40.6(13.2) | 40.5(12.9) | P<0.001 |
| Education respondent |  |  | P<0.001 |
| No education | 2416(19.9) | 9730(80.1) |  |
| Primary education | 544(10.7) | 4543(89.3) |  |
| Secondary education | 353(8.3) | 3881(91.7) |  |
| Higher education | 40(6.0) | 624(94.0) |  |
| Education partner |  |  | P<0.001 |
| No education | 1987(20.7) | 7624(79.3) |  |
| Primary education | 581(12.6) | 4048(87.4) |  |
| Secondary education | 507(9.5) | 4813(90.5) |  |
| Higher education | 170(9.4) | 1640(90.6) |  |
| Place of residence |  |  | P<0.001 |
| Urban | 618(12.4) | 4365(87.6) |  |
| Rural | 2735(16.0) | 14413(84.0) |  |
| Religion |  |  | P<0.001 |
| Catholic | 118(7.0) | 1577(93.0) |  |
| Other Christian | 483(7.4) | 6045(92.6) |  |
| Islam | 2648(19.9) | 10662(80.1) |  |
| Traditionalist | 84(18.9) | 360(81.1) |  |
| Other | 1(4.4) | 22(95.6) |  |
| Wealth Index |  |  | P<0.001 |
| Poorest | 1364(20.5) | 5279(79.5) |  |
| Poorer | 942(16.1) | 4920(83.9) |  |
| Middle | 543(12.7) | 3745(87.3) |  |
| Richer | 352(10.7) | 2943(89.3) |  |
| Richest | 152(7.4) | 1891(92.6) |  |
| Ethnicity |  |  | P<0.001 |
| Ekoi | 18(5.1) | 334(94.9) |  |
| Fulani | 643(26.5) | 1787(73.5) |  |
| Hausa | 1349(20.4) | 5275(79.6) |  |
| Ibibio | 21(7.4) | 263(92.6) |  |
| Igala | 17(7.3) | 215(92.7) |  |
| Igbo | 147(7.9) | 1708(92.1) |  |
| Ijaw/ Izon | 30(5.2) | 544(94.8) |  |
| Kanuri/ Beriberi | 215(26.9) | 584(73.1) |  |
| Tiv | 50(7.4) | 626(92.6) |  |
| Yoruba | 236(10.9) | 1934(89.1) |  |
| Others | 610(10.2) | 5400(89.8) |  |
| State of residence |  |  | P<0.001 |
| Akwa Ibom | 25(7.3) | 316(92.7) |  |
| Anambra | 12(4.6) | 248(95.4) |  |
| Bauchi/Gombe | 444(22.3) | 1544(77.7) |  |
| Edo | 26(6.8) | 357(93.2) |  |
| Benue | 67(8.9) | 683(91.1) |  |
| Borno | 232(25.1) | 693(74.9) |  |
| Crossriver | 26(5.9) | 417(94.1) |  |
| Adamawa | 107(13.1) | 709(86.9) |  |
| Imo | 11(5.0) | 208(95.0) |  |
| Kaduna | 78(10.2) | 688(89.8) |  |
| Kano | 213(18.1) | 963(81.9) |  |
| Katsina | 203(17.9) | 929(82.1) |  |
| Kwara | 58(13.5) | 372(86.5) |  |
| Lagos | 27(7.1) | 354(92.9) |  |
| Niger | 77(9.2) | 762(90.8) |  |
| Ogun | 59(14.3) | 355(85.7) |  |
| Ondo/ Ekiti | 62(8.2) | 690(91.8) |  |
| Oye | 37(9.1) | 370(90.9) |  |
| Nassarawa/ Plateau | 80(6.9) | 1083(93.1) |  |
| Rivers/ Bayelsa | 41(6.1) | 631(93.9) |  |
| Sokoto/ Zamfara | 478(26.5) | 1323(73.5) |  |
| Abia | 22(8.1) | 251(91.9) |  |
| Delta | 40(10.7) | 335(89.3) |  |
| Enungu/ Ebonyi | 88(10.6) | 741(89.4) |  |
| Jigawa | 204(21.2) | 760(78.8) |  |
| Kebbi | 154(21.5) | 563(78.5) |  |
| Kogi | 34(8.8) | 352(91.2) |  |
| Osun | 55(13.8) | 343(86.2) |  |
| Taraba | 88(11.6) | 674(88.4) |  |
| Yobe | 277(27.2) | 741(72.8) |  |
| Abuja | 28(8.0) | 323(92.0) |  |

*Data are expressed as mean (standard deviation) or as percentages.

^†^*P*-values for comparison between underweight and normal-weight subjects.
